# Guhong Injection Prevents Ischemic Stroke-Induced Neuro-Inflammation and Neuron Loss Through Regulation of C5ar1

**DOI:** 10.3389/fphar.2022.818245

**Published:** 2022-03-21

**Authors:** Jingjing Zhang, Rui Zhou, Guangzhao Cao, Yi Zhang, He Xu, Hongjun Yang

**Affiliations:** ^1^ Institute of Chinese Materia Medica, China Academy of Chinese Medical Sciences, Beijing, China; ^2^ Chinese Institute for Brain Research, Beijing, China; ^3^ College of Traditional Chinese Medicine, Guangzhou University of Chinese Medicine, Guangzhou, China; ^4^ Experimental Research Center, China Academy of Chinese Medical Sciences, Beijing, China

**Keywords:** stroke, safflower extract and aceglutamide, RNA-seq, inflammation, C5aR1

## Abstract

C5ar1 (CD88) has been identified as an important potential therapeutic target for regulating inflammation in ischemic stroke. In this study, the neuroprotective effect of Guhong injection (GHI) on middle cerebral artery occlusion (MCAO)-induced reperfusion injury was assessed and the mechanism was explored by RNA-seq technology. GHI administered for 6 consecutive days significantly decreased body weight loss, infarction rate, neurological deficient scores, and neuron loss but improved rat survival percentage and regional cerebral blood flow after MCAO surgery. Furthermore, we identified inflammation as a vital process and C5AR1 as a vital target in GHI-mediated protection by using RNA-seq analysis. Further experiments confirmed that GHI decreased C5AR1, C5A, CASP3, 8-OHdG, and inflammatory factors such as IL-1β, TNF, IL6, ICAM-1, MMP9, and MCP-1, and enhanced the expression of TIMP1, JAM-A, and laminin. Furthermore, GHI and its major components hydroxysafflower yellow A (HSYA) and aceglutamide (AG) enhanced cell viability and reduced LDH level and C5AR1 expression in a C5A-induced Neuro-2a cell damage model. In general, this study elucidated the mechanism of GHI against ischemic stroke by inhibiting inflammation and highlighted the potential important role of C5AR1 in ischemic stroke. This research provided new insights into the mechanism of GHI in resisting ischemic stroke and benefits of its clinical application.

## Introduction

Ischemic stroke, accounting for approximately 80% of all kinds of stroke, is a severe cerebral vascular disease that causes high disability and mortality in patients worldwide (Minnerup et al., 2012; Krishnamurthi et al., 2013). Due to the lack of blood supply, the ischemia causes irreversible injury such as neuron death in the brain ischemic core area while the neurons living in the penumbra area have the opportunity to be restored by timely reperfusion and drug administration. Importantly, recombinant tissue-type plasminogen activator (rtPA)-based reperfusion of the blood supply usually exaggerates the ischemic injury and leads to cerebral ischemia/reperfusion injury (IRI) (Chamorro et al., 2016) Thus, it is of importance to search for an effective therapy for salvaging the ischemic penumbra to rescue dying neuron cells and prevent reperfusion-induced damage.

Numerous clinical and experimental research articles have shown that reperfusion after ischemic stroke triggers immune disorders and inflammation which results in a detrimental effect on the rescue and recovery of brain ischemia (Petrovic-Djergovic, 2016). Notably, it has been proved that ischemic stroke initiates the occurrence of inflammation, while inflammation can also aggravate cerebral ischemia. At the onset of ischemic stroke, a large number of cells residing in the brain are activated in response to ischemia such as microglia, neutrophils, T cells, monocytes, and dendritic cells, which initiate inflammation and promote acute primary brain injury (Petrovic-Djergovic, 2016). As an important part of the innate immune system, the complement cascade plays an important role in pathogen recognition and response and is activated to aggravate inflammation and injury after cerebral ischemia (Ducruet et al., 2012). This inflammation in the brain exacerbates brain damage through enhancing oxidative damage, blood-brain barrier (BBB) impairment, energy metabolism disorder, and excitotoxicity, which will conversely worsen the recovery of clinical stroke patients (Shi et al., 2019). For example, the leakage of the BBB promotes the adhesion and infiltration of peripheral leucocytes and the infiltrated leucocytes further boost the BBB breakdown through increasing the synthesis of inflammatory factors and the release of matrix metalloproteinases (Neumann et al., 2015). Apart from that, the acute inflammation in the local brain will develop into a long-term inflammation in the global brain and persistently affect the recovery and restoration of neurological function in stroke patients (Pappata et al., 2000; Gerhard et al., 2005). Thus, targeting inflammation in ischemic stroke can block the following cascade damage in a timely manner and would be a beneficial treatment for ischemic stroke in the long-run.

Guhong injection (GHI), also named safflower extract and aceglutamide, is a standardized product recorded in the Chinese Pharmacopeia and is widely used for various cerebrovascular diseases, especially for ischemic stroke. Recent research has reported that GHI exhibited a remarkably neuroprotective effect in ischemic stroke (Zhang and Ning, 2015; Ai et al., 2017). In our previous research, administration of GHI and its two components immediately at the beginning of ischemic reperfusion prevented oxidative damage and cell apoptosis via enhancing the three antioxidant systems of Nrf2, Trx, and GSH and obviously promoted neuroprotection in ischemic stroke (Zhang et al., 2020a). Notably, the disease severity and prognosis of ischemic stroke is closely associated with inflammation (Pappata et al., 2000; Gerhard et al., 2005). GHI and its component, hydroxysafflower yellow A, was reported to intervene in inflammation to attenuate ischemic damage (Ye and Gao, 2008; Ai et al., 2017). However, the mechanism of GHI in inhibiting inflammation remains unclear. RNA-seq technology, which is characterized with high sensitivity, high accuracy, and high coverage, is a suitable method to systematically explore the mechanism of multi-component and multi-target traditional Chinese medicine (TCM) (Zhang et al., 2020b; Xiang et al., 2021). Applying this technology, Huangbai liniment was identified to activated Nrf2 and its downstream antioxidant genes to facilitate wound healing (Zhang et al., 2020b) while the Yixin-Shu capsule was identified to upregulate Trx2 and inhibit JNK/p38 activation to halt heart failure (Xiang et al., 2021). In this study, the potential mechanism of GHI against ischemic stroke was comprehensively and systematically explored by taking advantage of RNA-seq technology.

## Materials and Methods

### Animal Models and Drug Administration

Male 280–320 g Sprague-Dawley rats were purchased from Peking University Health Science Center. All animal experiments such as surgery, drug administration, and tissue harvesting were carried out according to the guidelines from the Committee on Animal Care and Use of the Institute of Chinese Materia Medica, China Academy of Chinese Medical Sciences. The rats were kept in a house with free access to water and food in a 12-h light/dark cycle environment. Tonghua Guhong Pharmaceutical Co., Ltd. provided the GHI for the following experiments. The major compounds of SAAG were quantified by high-performance liquid chromatography (HPLC) and syringing, including hydroxysafflower yellow A, aceglutamide, anhydrosafflower yellow B, adenosine, uridine, and guanosine.

After anesthetization with sodium pentobarbital (i.p.), the rats underwent middle cerebral artery occlusion (MCAO) for 1.5 h, followed by 6 days of reperfusion. Specifically, the middle cerebral artery was occluded by inserting 4–0 nylon thread into the internal carotid artery (ICA) via the right common carotid artery (CCA) to block the origin of the artery, and 1.5 h later, the thread was taken away to initiate reperfusion. The whole surgery was performed at 37°C to maintain the rats’ body temperature. The rats in the sham group were subjected to the same operation process except for inserting the thread. The rats subjected to MCAO surgery were randomly assigned into four groups from a random number table including an ischemia/reperfusion group (I/R, physiological saline, i.p.), I/R + GL group (1.25 ml·kg^−1^, i.p.), I/R + GH group (5 ml·kg^−1^, i.p.), and I/R + Ginaton group (8 ml·kg^−1^ ginaton; i.p.). Those rats subjected to MCAO surgery that did not display ischemia in the brain were removed from the experiments. The dose of GHI was determined by considering the equivalent dose conversion from the clinical dose, our previous research, and our pre-experiment results. Both GHI and ginaton were administrated by intraperitoneal (i.p.) injection at 6 h after reperfusion and the rats were then injected daily with these drugs for 6 days, respectively. Finally, the rats were anesthetized and sacrificed and the serum and brain tissue of the rats were collected for the next experiments.

### Weight Change and Survival Percentage

The weight of the SD rats treated with various drugs was evaluated before and after MCAO surgery. And the weight change was calculated by using the following: ΔBody Weight = The weight of SD rats after MCAO surgery-the weight of SD rats before MCAO surgery. The survival percentage was also calculated as the following formula: survival percentage= (The number of overall rats–the number of survival rats)/the number of overall rats*100%.

### Longa 5 Neurological Deficit Scores and TTC Staining

Longa 5 neurological deficient scores (Longa et al., 1989) and 2,3,5-Triphenyltetrazolium chloride (TTC) staining were applied to evaluate the protection of GHI against MCAO-induced injury. A slight modification was used in using the Longa 5 method. For example, the situation between score 1 and score 2 was recorded as 1.5. As for TTC staining, the brain tissue was separated into brain slides with a thickness of 2 mm, and then the slides were incubated with 2% (w/v) TTC at 37°C for 20 min and then fixed with paraformaldehyde. The infarction area was white while the normal area in the brain tissue was stained red. The infarction rate of the brain after various treatments was calculated with the following formula: Infarction rate = the infarcted area of the brain/the overall area of the brain. After anesthetization with sodium pentobarbital (i.p.), the rats subjected to MCAO surgery and treated with GHI for 6 consecutive days were used for reginal cerebral blood flow (rCBF) detection. The rCBF in the right hemisphere of the rats was measured by applying laser Doppler flowmetry and qualified with the related analysis system.

### Hematoxylin and Eosin (H&E) and Nissl Staining

Polyformaldehyde (4% (v/v)) was used to fix the harvested brain tissues. Then the brain tissue was dehydrated with ethanol and then embedded using paraffin. Then the embedded brain tissue was cut into sections with 5 μm thickness for H&E and Nissl staining. Hematoxylin was used for nuclei staining while eosin was used for cytoplasm and extracellular matrix staining. As for Nissl staining, the sections were dewaxed and stained with 1% tar violet or 1% thionine to detect neuron damage. The density of neuron and Nissl bodies was quantified with ImageJ software.

### RNA-Seq Analysis and Network Construction

The RNA of the brain samples was extracted using TRIzol reagent. After the quality of RNA was evaluated by using an RNA Nano 6000 Assay Kit, the purification of mRNA by poly-T oligo-attached magnetic beads was performed. Divalent cations were used to fragment the mRNA with First Strand Synthesis Reaction Buffer (5X). First-strand cDNA synthesis was conducted by M-MuLV Reverse Transcriptase (RNase H-) and a random hexamer primer. The synthesis of second-strand cDNA was conducted with RNase H and DNP Polymerase I. After adenylating the 3’ ends in the DNA fragment, the ligation of the adaptor with a hairpin loop structure was performed for hybridization. The cDNA fragments with 370–420 bp were purified by using the AMPure XP system. The PCR experiment was finished by using Phusion High-Fidelity DNA polymerase and then purification of PCR products was conducted. Finally, the Agilent Bioanalyzer 2100 system was used for evaluation of the library quality.

The index-coded samples were clustered with the cBot Cluster generation system through TruSeq PE luster Kit V3-cBot-HS (Illumia). The sequencing of the established library was performed with the Illumina Novaseq platform and the paired-end reads at 150 bp were obtained. The rat reference genome with ensemble release 91 was used for gene mapping while differentially expressed genes (DEGs) were analyzed by the DEseq2 R package (1.20.0). The RNA-seq technology process including RNA extraction, gene sequencing, and mapping was accomplished by Novogene Bioinformatics Technology Co., Ltd. (Beijing, China) (Zhang et al., 2017; Zhang et al., 2017; Zhou et al., 2020). And the raw data of the RNA-seq results have been uploaded into https://www.ncbi.nlm.nih.gov/bioproject/736751. The genes with |log2 Fold change|≥1 and *p* value < 0.05 between the two treatments were identified as DEGs. And the enrichment of DEGs between I/R + GH and I/R was performed by Metascape (https://metascape.org/gp/index.html#/main/step1). The MCAO surgery-induced DEGs, reversed by GHI, were used for network construction through Cytoscape v3.4.0.

To demonstrate the relationship of the three previously published antioxidant systems and C5AR1-mediated inflammation in this research, the reversed inflammation-related targets such as IL-1β, TNF, IL6, ICAM-1, MMP9, MCP-1 (also named CCL2), Timp1, C5A, and C5AR1 in this study and the restored targets in the three antioxidant systems including Nrf2, NQO1, PRX2, TrxR, GSH, GR, GCLM, GSH-PX, and glutathione reductase (GR) in a previous study (Zhang et al., 2020a) were combined and the association network was constructed through the String database.

### Enzyme-Linked Immunosorbent Assay

The protein extracted from the rat brain was quantified by using a BCA Protein Assay Kit (ab102536). MCP-1 (SEA087Ra), IL-6 (SEA079Ra), MMP9 (SEA553Ra), TIMP1 (SEA552Ra) from Cloud-clone corp. Wuhan, IL-1β (3001/1), TNF (2784/1), ICAM-1 (1121/2) from Beijing North Institute of Biotechnology Co., Ltd., C5AR1 (MM-70648R, MM-44670M) from Jiangsu Feiya Biotechnology Co., Ltd., and C5A (E-EL-R3011) from Elabscience Biotechnology Co., Ltd. were used for ELISA measurement. Specifically, the ELISA experiments were performed according to the procedures in the kits’ instructions. The extracted protein sample was added into the plates which were immobilized with corresponding specific antibodies, then the plates were washed with PBS five times. After that, the secondary antibody which was conjugated with a horseradish peroxidase was added for incubation before being measured under a microplate reader (DNM-9602G).

### Immunofluorescence Staining

Polyformaldehyde (4% (v/v)) was used to fix the harvested brain tissues. Then the brain tissue was dehydrated with ethanol and then embedded by using paraffin. Then the embedded brain tissue was cut into sections with 5 μm thickness for IF staining. Specifically, the sections were incubated with 0.5% Triton X-100 for 30 min, then blocked with bovine serum albumin. After that, a primary antibody was loaded overnight at 4°C, and then followed by second antibodies which were conjugated with Alexa Fluor 488 or Alexa Fluor 647. Finally, the nuclei were stained with 4′,6-diamidino-2-phenylindole (DAPI, Sigma-Aldrich) for 20 min at room temperature before observation under an LSM-880 confocal microscope (Carl Zeiss, Oberkochen, Germany). The antibodies used in this manuscript were C5AR1 (SC53795), 8-OHdG (SC-66036), MMP9 (ab76003), junctional adhesion molecule 1/JAM-A (ab180821), laminin receptor (ab133645), cleaved caspase-3 (CST, 9664S), 8-OHdG (sc-66036), Neuron (ab178847), and Iba1 (ab177487).

### CASP3 Activity Evaluation

The protein sample was harvested from the rat brain and then quantified by a BCA Protein Assay Kit (ab102536). CASP3 activity was measured by applying the assay kits (G015-2) from Nanjing Jiancheng Bioengineering Institute. Briefly, the harvested protein extraction was incubated for 1.5 h at 37°C with 25 μg of Ac-DEVD-pNA, which was recognized by CASP3 as a substrate. And the absorbance was detected through a microplate reader (Molecular Device, United States) at the wavelength of 405 nm. CASP3 activity indicated the amount of enzyme cleaving each nM of Ac-DEVD-pNA per hour at 37°C.

### Cell Experiments

National Infrastructure Cell Corporation provided mouse neuroblastoma N2a (Neuro-2a) cells for *in vitro* experiments. The cells were cultured in DMEM (Gibco) with 10% FBS (Gibco) and 100 U/ml of penicillin/streptomycin in a humidified incubator with 5% CO_2_ at 37°C. In addition, Neuro-2a cells were incubated with recombinant human C5A protein (ab167724) for 48 h to establish a C5A-induced cell damage model. National Institutes for Food and Drug Control provided hydroxysafflower yellow A (HSYA) and aceglutamide (AG). First of all, Neuro-2a cells were treated with various concentrations of GHI, HSYA, and AG, but without C5A for 48 h to investigate their safe concentrations. To evaluate the therapeutic effect, cells were treated with 10^–4^ μg/ml of recombinant human C5A protein and then treated with GHI, HSYA, and AG for 48 h to investigate cell viability and lactate dehydrogenase (LDH) level. CCK-8 with a microplate reader (Molecular Devices, United States) was used for detecting cell viability. Furthermore, the expression levels of LDH (A020) and C5AR1 (MM-44670M) were detected by the kit after recombinant human C5A proteins were treated with GHI (10 μl/ml), HSYA (10 μg/ml), and AG (5 μg/ml) for 48 h.

### Statistical Analysis

**FIGURE 1:**
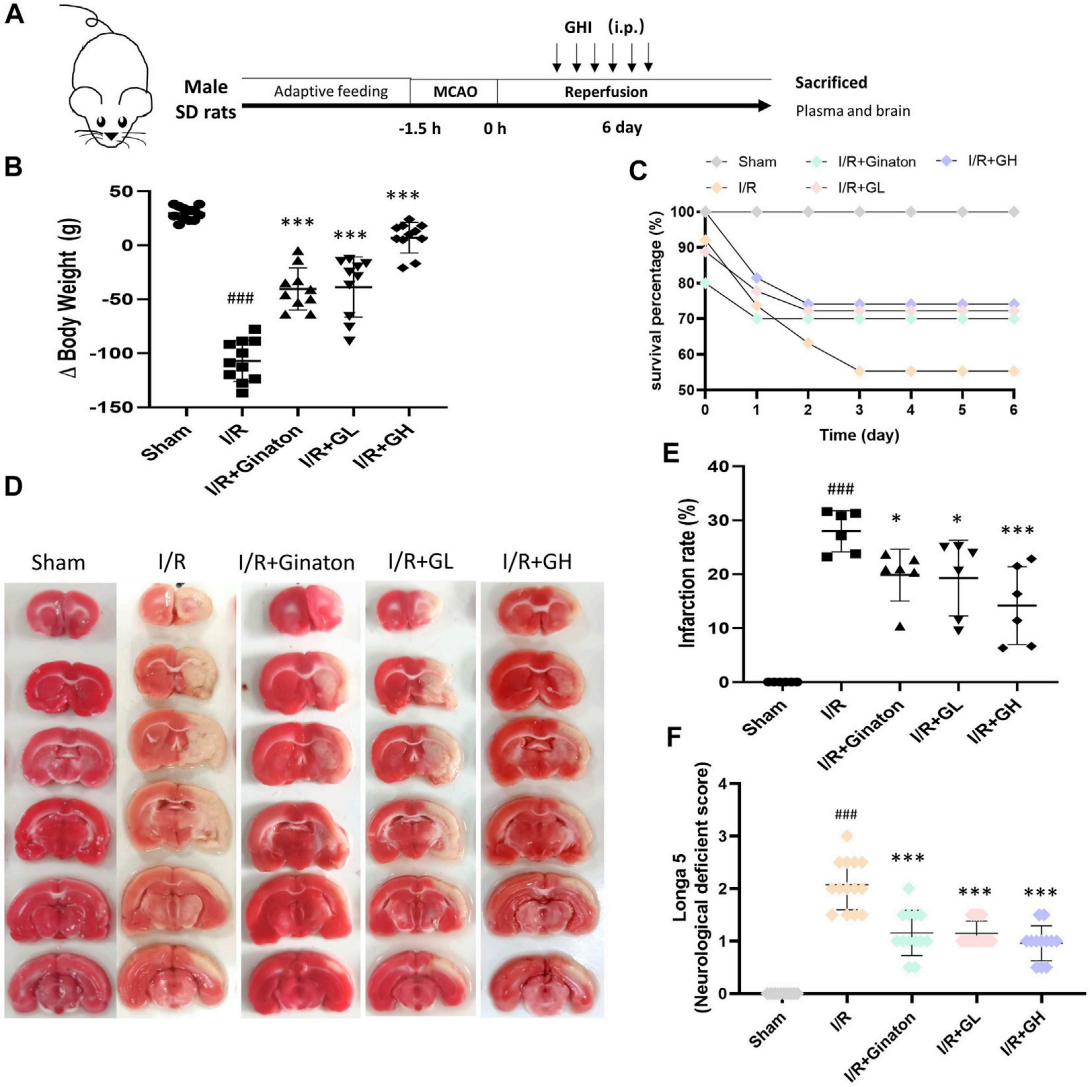
GHI protected against MCAO-induced I/R injury at day 6. **(A)** Schematic diagram of MCAO-induced rat model treated with GHI; **(B)** Δbody weight (*n* = 10–12); **(C)** Survival percentage (*n* = 16–32); **(D)** TTC staining representative images; **(E)** the qualified infarction ratio through TTC staining by using ImageJ (*n* = 6); **(F)** Longa 5 neurological deficient scores (*n* = 12); ^###^ *p* < 0.001 compared with sham, *p < 0.05, ***p < 0.001 compared with the I/R group.

The data were analyzed using SPSS and presented as the means±standard deviations. The data were analyzed by one-way ANOVA with SPSS 22.0 software. Before multiple comparisons between the means of each group, a Levene test was used to test the homogeneity of variance. If the variance was homogeneous (*p* > 0.05), an LSD test was used to analyze the data. If the variance was uneven (*p* < 0.05), Dunnett’s T3 test was used to analyze the data. Neurological deficit scores in Figure 1 were analyzed by using the Kruskal–Wallis test. The Chi-square test was used for statistical analysis of cell viability results. Significance was labeled as follows: ^#^ *p* < 0.05, ^##^ *p* < 0.01, and ^###^ *p* < 0.001 compared to the sham group; *p < 0.05, **p < 0.01, and ***p < 0.001 compared to the I/R group.

## Result

### GHI Obviously Protected Against MCAO-Induced Brain Damage

**FIGURE 2:**
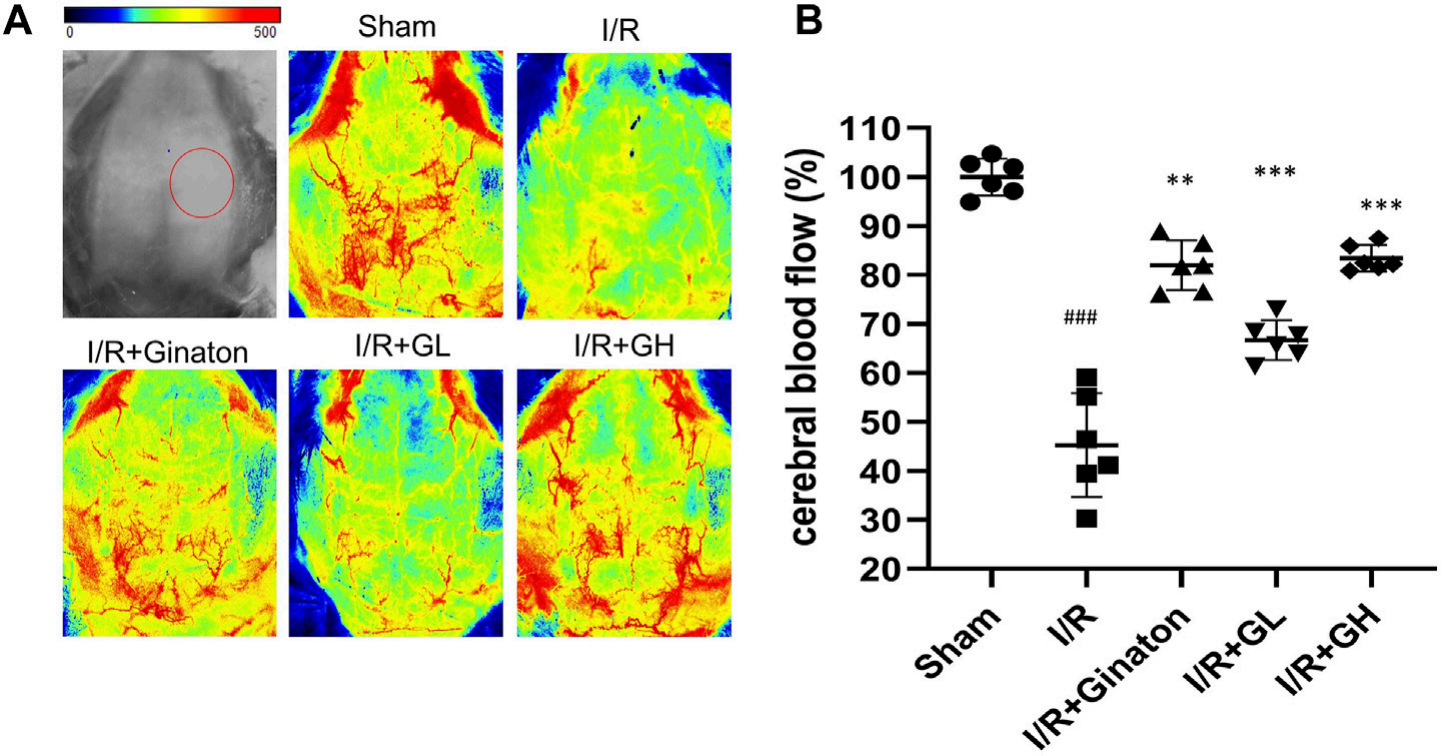
Administration of GHI for 6 consecutive days enhanced rCBF in MCAO-induced rats; **(A)** the representative rCBF images of rats with various treatments; **(B)** quantitative results of rCBF (*n* = 6); ^###^ *p* < 0.001 compared with sham, **p < 0.01, ***p < 0.001 compared with the I/R group. RNA-seq analysis of GHI-mediated cerebral protection.

To evaluate the protection of GHI on MCAO-induced cerebral injury, rat weight change, survival percentage, infarction rate, and Longa 5 neurological deficient scores were determined. MCAO surgery led to obvious weight loss and a reduction of survival percentage, whereas GHI and ginaton markedly prevented weight loss and increased survival percentage (Figures 1B,C). Besides, the increased infarction rate and neurological deficit scores caused by MCAO surgery were significantly decreased by GHI and the protective effect was in a dose-dependent manner (Figures 1D–F). The protection of GHI at 5 ml/kg on MCAO-induced injury was comparative to that of ginaton as evidenced by the reduction in weight loss, infarction rate, and neurological deficient scores, and the increase of survival percentage when compared to the I/R group (*p* < 0.05, as indicated by Figure 1). The rCBF was detected to evaluate the effect of GHI on the restoration of cerebral blood flow after IRI. As shown in Figure 2, MCAO surgery caused an obvious decrease in rCBF in the I/R group, whereas this was significantly improved by GHI treatment.

### RNA-seq Analysis of GHI-Mediated Cerebral Protection

**FIGURE 3:**
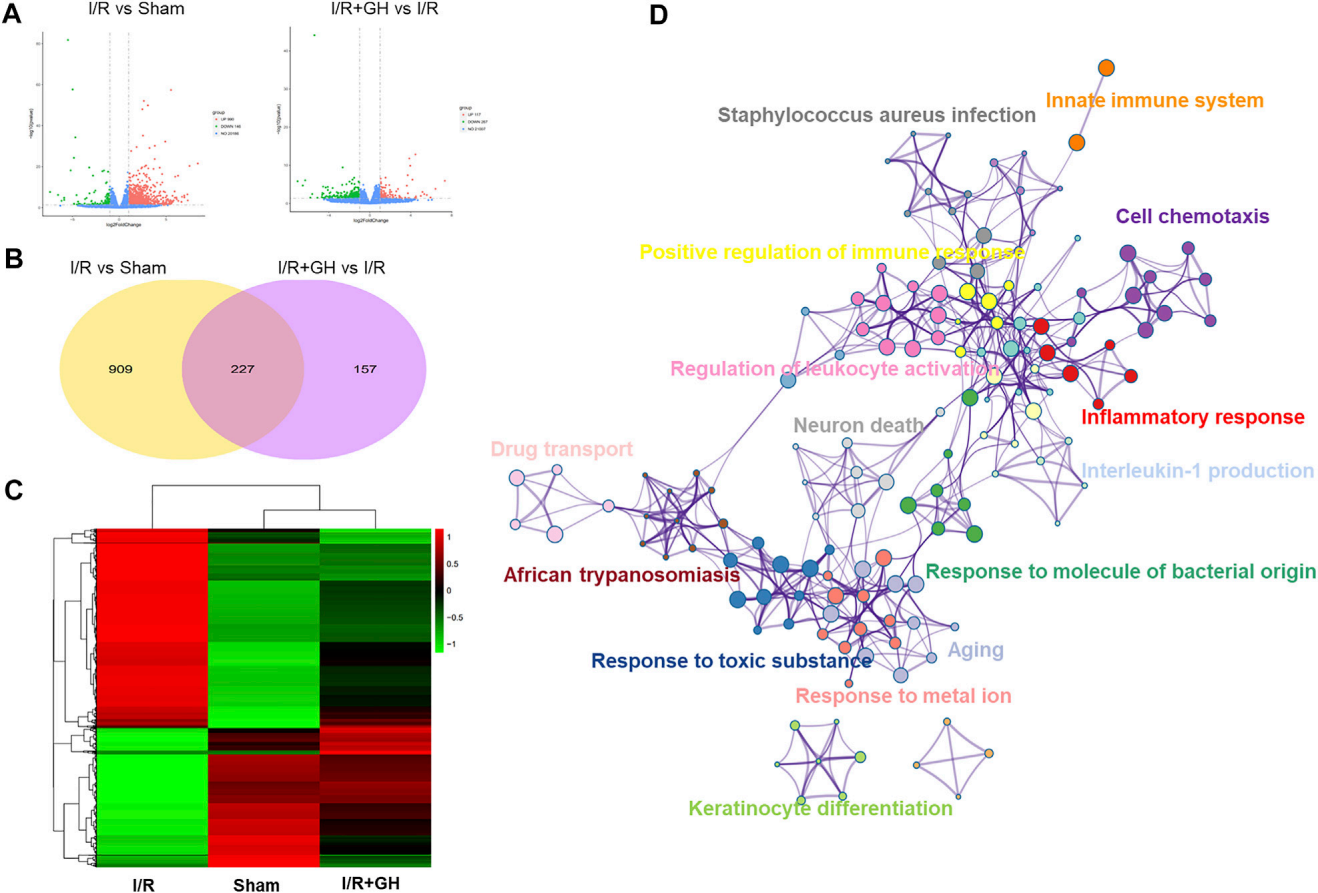
The global gene expression of GHI-mediated protection against I/R by RNA-seq analysis; **(A)** volcano plot (*n* = 3); **(B)** Venn diagram; **(C)** hierarchical heatmap; and **(D)** GO enrichment of DEGs between I/R + GH and I/R using Metascape.

RNA-seq technology was applied to systematically explore the underlying mechanism of GHI against MCAO-induced cerebral injury. As shown in Figure 3A, 1136 differentially expressed genes (DEGs) including 990 upregulated and 146 downregulated genes were identified in the I/R group when compared to the sham group. In contrast to that in the I/R group, 384 DEGs including 117 upregulated and 267 downregulated DEGs were found in the I/R + GH group. And 227 overlapped DEGs were observed between the two treatments of I/R vs Sham and I/R+GH vs I/R (Figure 3B). The hierarchical heatmap demonstrated that gene expression after GHI treatment was more similar to that in the sham group when compared to that in the I/R group (Figure 3C). The DEGs between I/R + GH and I/R were enriched in inflammation-associated gene ontology (GO) terms such as “inflammatory response”, “cell chemotaxis”, “interleukin-1 production”, “positive regulation of immune response”, and “innate immune system”, and many other GO terms such as “neuron death”, “response to molecule of bacterial origin”, “response to toxic substance”, “response to metal ion”, and “drug transport” (Figure 3D). Thus, the preliminary analysis indicated that GHI may affect inflammation-related biological processes to prevent cerebral injury caused by MCAO.

### C5AR1 was Identified to be Vital in GHI-Mediated Cerebral Protection

**FIGURE 4:**
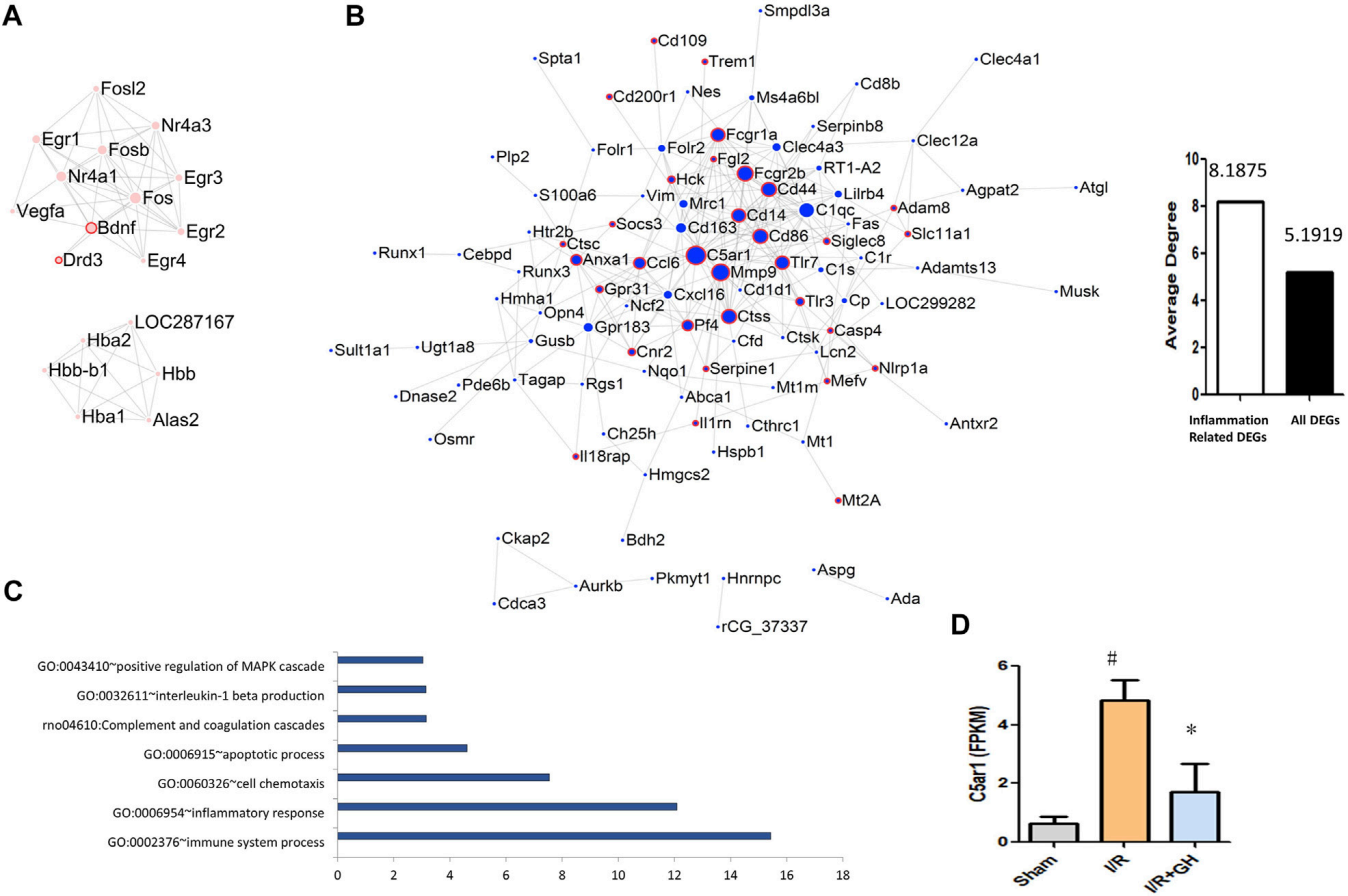
Further analysis and network of DEGs between I/R + GH and I/R. **(A)** The network of DEGs that were significantly downregulated after I/R treatment and were further significantly upregulated by GHI; **(B)** the network of DEGs that were significantly upregulated after I/R treatment and were further significantly downregulated by GHI, the red frame indicates that the DEGs were associated with the inflammation process; **(C)** the enriched biological process of the DEGs that were upregulated after I/R and then downregulated by GHI; and **(D)** the expression level of C5AR1 by RNA-seq identification ^#^ *p* < 0.05 compared with sham, *p < 0.05, compared with the I/R group. The expression of C5AR1 and C5A was reduced by GHI.

To further investigate the underlying mechanism of GHI-mediated protection, the DEGs identified after MCAO surgery was further significantly reversed by GHI, were used for the following analysis. As shown in Figures 4A,B, the network of the 50 reversed upregulated DEGs and 175 downregulated DEGs by GHI was constructed using Cystoscape. And the genes that were associated with inflammation processes were marked with a red frame. Among these 175 reversed downregulated DEGs, 32 genes were associated with inflammation processes. Importantly, the average degree of inflammation-related DEGs was higher than that of all DEGs in the network and DEGs with the top degree were C5AR1 and MMP9 (Figure 4B). The 175 reversed downregulated DEGs were enriched in GO terms including “GO: 0002376∼immune system process”, “GO: 0006954∼inflammatory response”, “GO: 0060326∼cell chemotaxis”, “GO: 0006915∼apoptotic process”, “rno04610: complement and coagulation cascades”, “GO: 0032611∼interleukin-1 beta production”, and “GO: 0043410∼positive regulation of MAPK cascade” (Figure 4C). The RNA-seq results indicated that GHI significantly decreased the expression of C5AR1 in I/R (Figure 4D).

### The Expression of C5AR1 and C5A was Reduced by GHI

**FIGURE 5:**
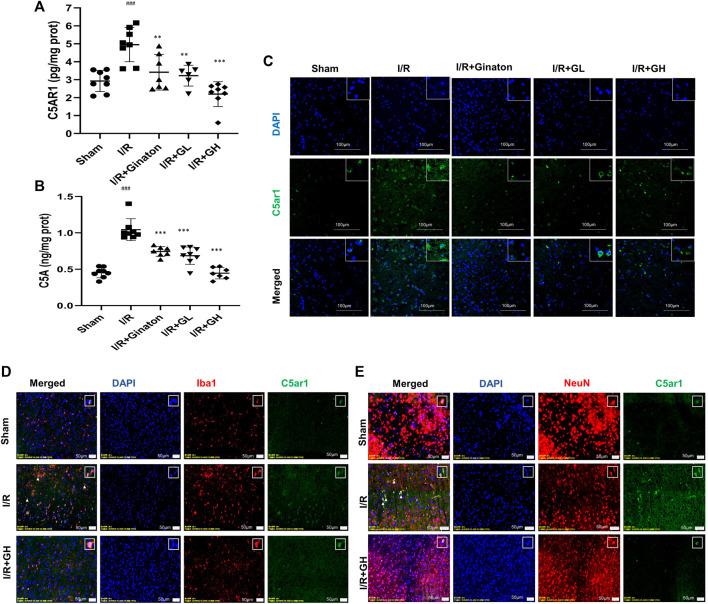
GHI reduced the expression of C5AR1 and C5A in the cortex. **(A–B)** ELISA of C5AR1 and C5A (*n* = 6–8); **(C)** IF for C5AR1 (green); **(D)** IF for C5AR1 (green) and Iba1 (red); **(E)** IF for C5AR1 (green) and NeuN (red); nuclei (blue). The white triangle indicates the expression of C5AR1 in neurons or microglia, scale bar: 100 μm. ^###^ *p* < 0.001 compared with sham, *p < 0.05, ***p < 0.001 compared with the I/R group. GHI decreased inflammatory cytokines but restored JAM-A and laminin expression.

To further confirm the network analysis results, the expression of C5AR1 was determined using ELISA and an IF experiment. C5aR is an important receptor of C5A, and the C5AR1-C5A axis is involved in neurodegenerative diseases and inflammation activation of various cell types. Consistent with the result from RNA-seq analysis, MCAO caused a sharp increase in C5AR1 expression, whereas it was significantly reduced by GHI treatment (Figure 5). GHI can also decrease ischemia-induced upregulation of C5A expression after MCAO surgery (Figure 5B). Besides, I/R caused the increase in the expression of C5AR1 in both neurons and microglia and these were obviously decreased by GHI treatment (Figures 5D,E).

### GHI Decreased Inflammatory Cytokines and Increased JAM-A and Laminin Expression

**FIGURE 6:**
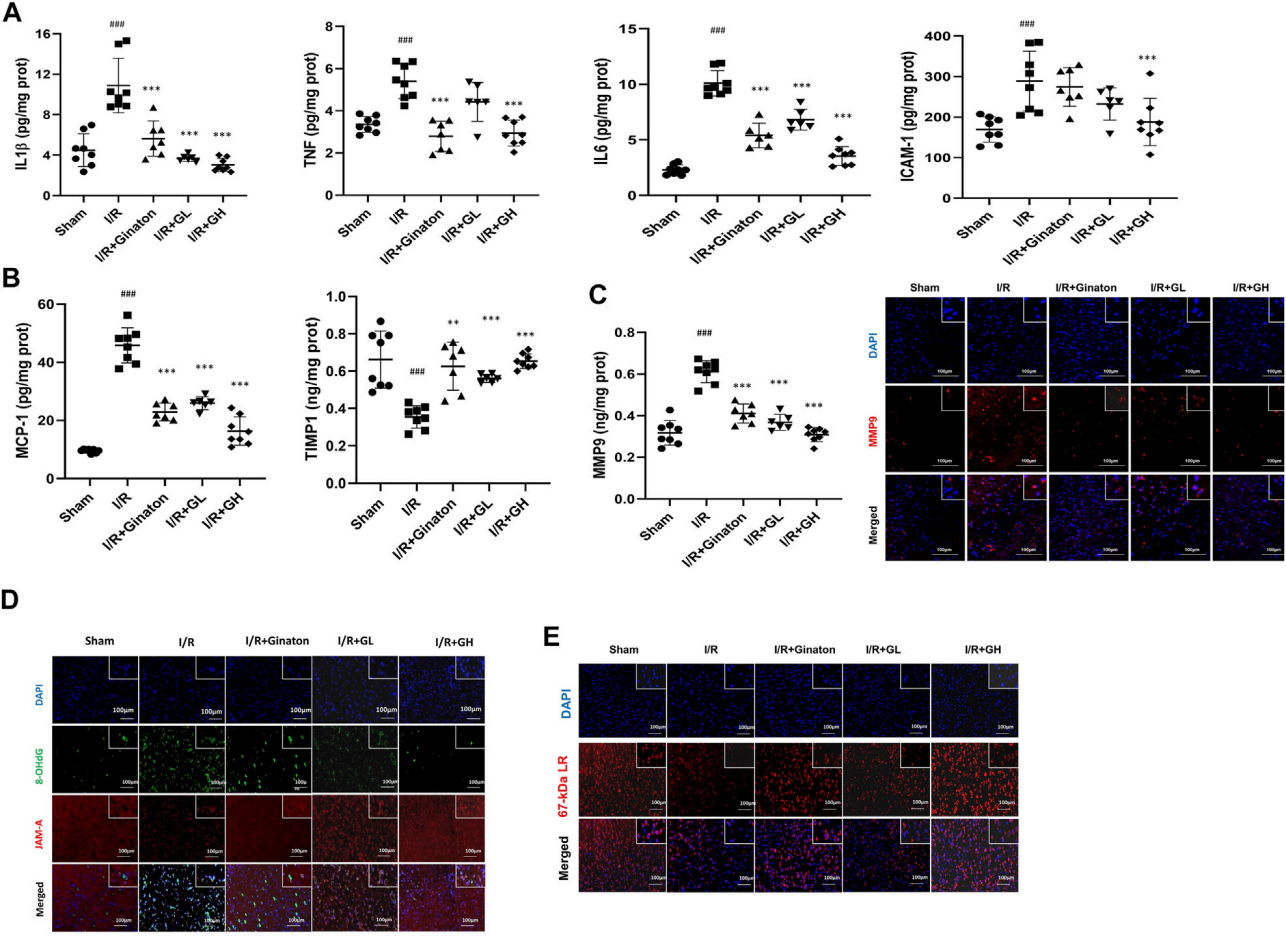
GHI inhibited inflammation and restored JAM-1 and laminin expression. **(A–E)** ELISA results of IL-1β, TNF, IL6, and ICAM-1 (*n* = 6–8); **(B)** ELISA results of TIMP1 and MCP-1 (*n* = 6–8); **(C)** ELISA results of MMP9 (*n* = 6–8) and IF for MMP-9 (red) in the cortex of rat brains, scale bar: 100 μm; **(D)** IF for JAM-A (red) and 8-OHdG (green) in the cortex of rat brains. **(E)** IF for laminin (red) in the cortex of rat brains; the nuclei are indicated in blue, scale bar: 100 μm, ^###^ *p* < 0.001 compared with sham, *p < 0.05, ***p < 0.001 compared with the I/R group. GHI prevented neuron loss in MCAO-induced injury.

MCAO-induced I/R injury caused a sharp increase of multiple inflammatory cytokines such as IL-1β, TNF, IL6, ICAM-1, MMP9, and MCP-1 (Figures 6A–E). In contrast, GHI treatment obviously decreased these inflammatory cytokines after IRI. Notably, ginaton treatment decreased IL-1β, TNF, IL6, MMP9, and MCP-1, but did not affect the ICAM-1 level in the rats with ischemic injury. TIMP1 is a metalloproteinase inhibitor of MMPs including MMP9 by binding to their catalytic zinc cofactor. As shown in Figure 6, MCAO-induced I/R led to a significant decrease in TIMP1 expression whereas this was reversed by the treatment of GHI or ginaton. JAM-A and laminin, which are important molecules in maintaining BBB integrity, were evaluated by IF. The weak immunostaining of JAM-A and laminin in the I/R group was enhanced by GHI treatment at day 6 and the enhancement was more prominent in the I/R + GH group than that in the I/R + GL group (Figures 6D,E).

### GHI Prevented Neuron Loss in MCAO-Induced Injury

**FIGURE 7:**
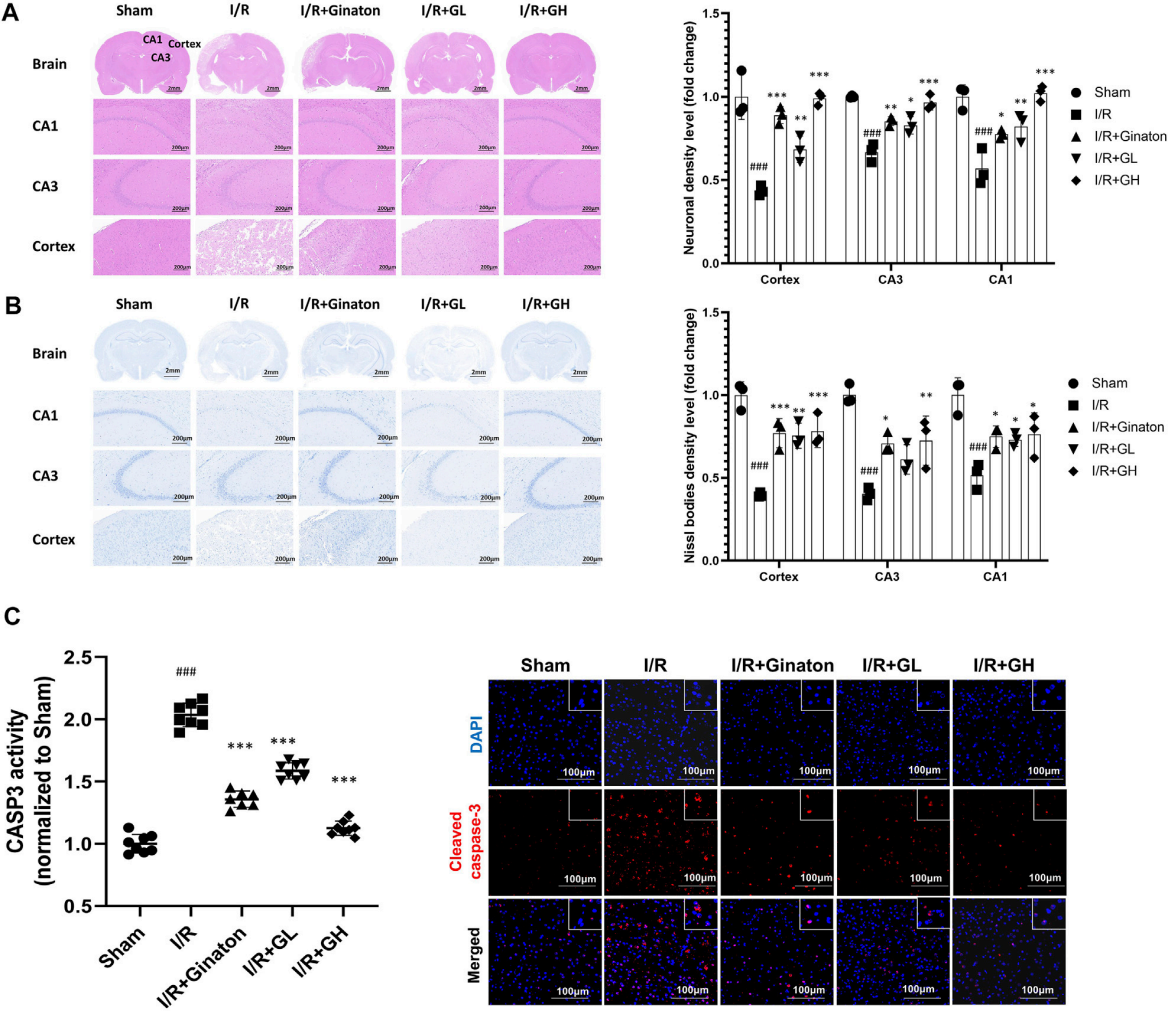
Administration of GHI for 6 days reduced neuron loss after IRI; **(A)** H&E staining and the relative density (fold change of sham) in the cortex and hippocampal CA1 and CA3 regions for each group (*n* = 3); **(B)** Nissl staining and Nissl bodies density level (fold change of sham) in the cortex and hippocampal CA1 and CA3 regions for each group (*n* = 3); **(C)** CASP3 activity (*n* = 6–8) and IF for cleaved CASP3 (red) ^###^ *p* < 0.001 compared with sham, *p < 0.05, ***p < 0.001 compared with the I/R group.

In this study, H&E and Nissl staining was used to evaluate the neuron loss in the cortex and CA1 and CA3 regions of the hippocampus. The majority of neurons in the I/R group showed pyknotic nuclei and low staining, indicating the injury of neurons in the ischemic region of the rat brain (Figure 7A). In contrast, the treatment of GHI significantly decreased the number of neurons with pyknotic nuclei in the cortex and CA1 and CA3 regions of the hippocampus in cerebral ischemia rats. In addition, Nissl staining indicated that the majority of neurons in the cortex and CA1 and CA3 regions of the hippocampus in the I/R group were shrunken and sparsely distributed with low staining, indicating an obvious loss of Nissl bodies in neurons (Figure 7B). However, GHI treatment for 6 days caused a strong staining and relatively dense, regular arrangement of neurons in these regions. Besides, I/R-treated rats demonstrated a higher expression and activity of cleaved CASP3, whereas this was reduced by the treatments of GHI or ginaton (Figure 7C). All these results showed a beneficial role of GHI in preventing neuron loss after IRI.

### GHI Inhibited C5A-Induced Neuro-2a Cell Damage

**FIGURE 8:**
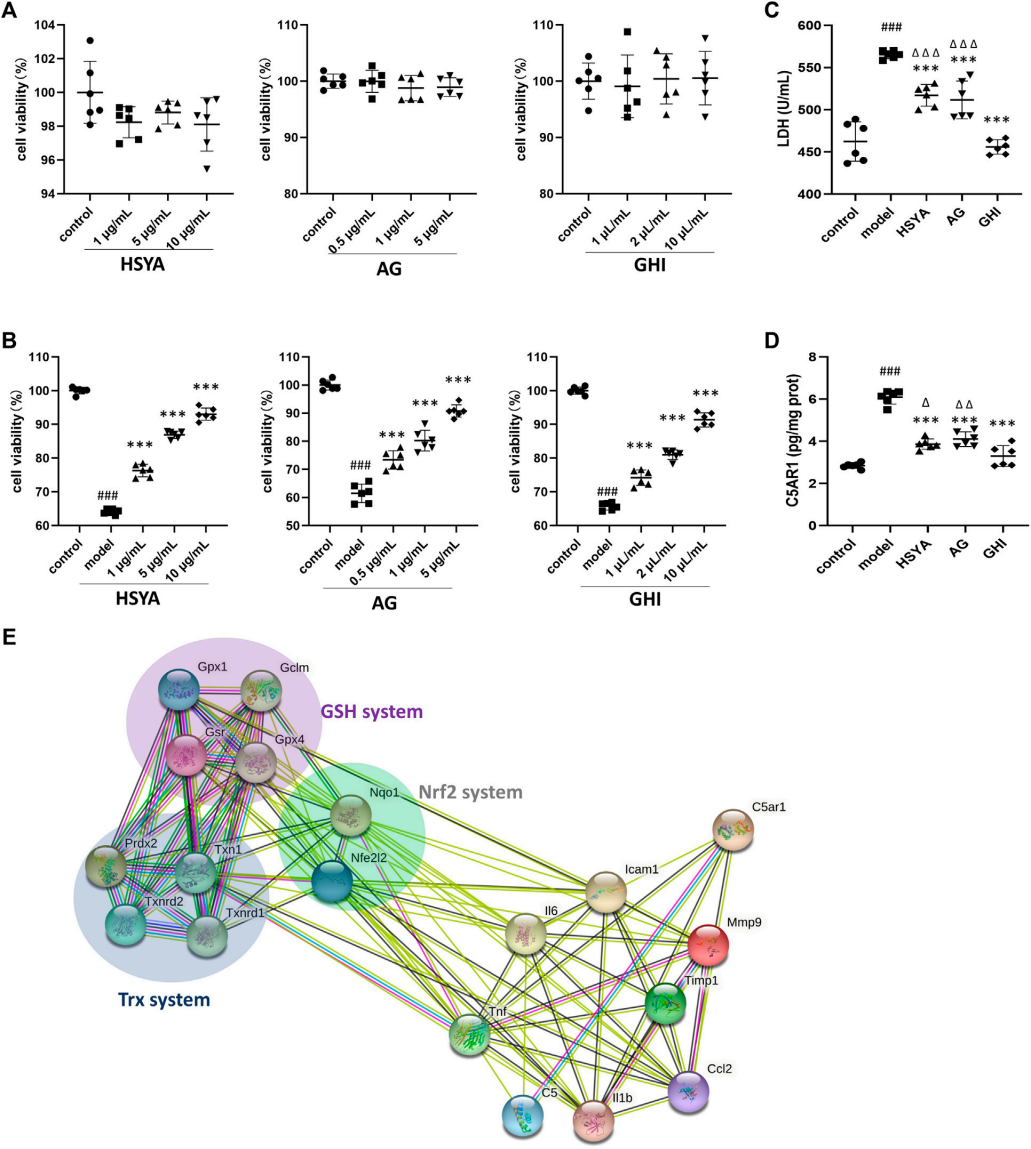
GHI, HSYA, and AG protected against C5a-induced neurotoxicity in Neuro-2a cells. **(A)** Viability of Neuro-2a cells only treated with HSYA, AG, and GHI (*n* = 6); **(B)** viability of Neuro-2a cells in the C5a-induced cell model (*n* = 6); **(C)** LDH levels (*n* = 6); **(D)** ELISA of C5AR1 (*n* = 6); **(E)** the association network of GHI-reversed targets in the three antioxidant systems and the reversed inflammation targets in this research by the String database. ^###^ *p* < 0.001 compared with control, ^***^ *p* < 0.001 compared with the model group, ^*p* < 0.05, ^ ^*p* < 0.01, and ^ ^ ^*p* < 0.001 compared with the GHI group.

To further confirm the mechanism of GHI, GHI and its two major components, HSYA and AG, were used to evaluate their effects on C5A-induced cell damage. As indicated in Figure 8A, pre-treatment of HSYA, AG, and GHI with Neuro-2a cells alone did not inhibit cell viability. Importantly, C5A stimulation significantly reduced cell viability, whereas this was ameliorated by HSYA, AG, and GHI (Figure 8B). In addition, exposure of Neuro-2a cells with C5A exhibited an obvious increase in LDH and C5AR1 levels which was significantly reversed by HSYA, AG, and GHI. Importantly, GHI demonstrated better cell protection than HSYA and AG in reducing LDH and C5AR1 levels (Figures 8C,D). Thus, GHI and its major components, HSYA and AG, decreased C5AR1 and protected neuron cells against C5A-induced injury.

Our previous research showed that administration of GHI as early as the onset of reperfusion, restored three antioxidant systems including Nrf2, GSH, and Trx at 48 h after the surgery, which prevented oxidative damage and cell apoptosis in ischemic stroke (Zhang et al., 2020a). In this study, the inhibition of inflammation and targeting C5AR1 was identified as the key process in the long-term repair of GHI in ischemic stroke. And multiple inflammation-related targets such as IL-1β, TNF, IL6, ICAM-1, MMP9, MCP-1 (also named CCL2), Timp1, C5A, and C5AR1 were reversed in this study (Figures 5, 6). To demonstrate the relationship of the three previously published antioxidant systems and C5AR1-mediated inflammation in this research, a network of reversed antioxidant targets and the reversed inflammatory factors in this study was established by using the String database. As indicated by Figure 8E, the GHI-reversed targets in the three antioxidant systems were closely associated with GHI-reversed inflammation targets.

## Discussion

Inflammation plays a vital role in ischemic stroke, which greatly affects disease severity and prognosis of patients with ischemic stroke (Pappata et al., 2000; Gerhard et al., 2005). After cerebral ischemia reperfusion, continuous administration of GHI for 6 days significantly decreased weight loss, infarction rate, and neurological deficient scores, and also improved rCBF and survival percentage in the rats subjected to the MCAO operation. The RNA-seq systematic exploration indicated that the inflammatory response with C5AR1 as a core target was important in GHI-mediated neuroprotection. *In vivo*, GHI decreased C5AR1, C5A, and multiple inflammation-related targets including IL-1β, TNF, IL6, ICAM-1, MMP9, MCP-1, and Timp1 in MCAO-induced brain injury. To prove the vital role of C5AR1, C5A was used to induce the Neuro-2a cell damage model, and GHI remarkably enhanced cell viability and decreased LDH level in the C5A-induced cell damage model. Additionally, the major components in GHI, HSYA, and AG obviously increased cell viability and decreased C5AR1 expression *in vitro*.

Inflammation acts as a central role in the pathogenesis of ischemic stroke including its occurrence, development, and recovery (Gerhard et al., 2005; Petrovic-Djergovic, 2016)). Importantly, cerebral injury can be aggravated by inflammation after ischemic stroke, which worsens the prognosis of clinical stroke patients (Shi et al., 2019). Once ischemic stroke is initiated, microglia activation obviously leads to reactive oxygen species (ROS) overproduction and cytokine secretion, which causes cell loss and BBB disruption in the brain. The produced cytokines such as IL-1β, TNF, MMP9, and MCP-1 cause direct and indirect damage in brain structures and neural cells (Seifert et al., 2014) (Candelario-Jalil et al., 2009) (Latour et al., 2004). In this study, multiple inflammation-related GO terms were affected by GHI and many inflammation-related targets including C5AR1, C5A, IL-1β, TNF, IL6, ICAM-1, MMP9, and MCP-1 were reduced by GHI. All these results highlighted the important role of inflammation inhibition in GHI-mediated protection. In addition to inflammation, ROS damage and cell apoptosis as well as BBB impairment intervened with each other and contributed to aggravate injury after reperfusion in ischemic stroke. In this study, inhibition of cell apoptosis and dysfunction in BBB proteins were found after GHI treatment, as shown by the reduction of CASP3 expression and activity, restoration of laminin and JAM-A proteins, and the decrease of the 8-OHdG level. Additionally, GHI-mediated targets in three antioxidant systems such as Nrf2, GSH, and Trx at 48 h after reperfusion (Zhang et al., 2020b) were closely associated with the reversed inflammation targets in this study, indicating a tight relationship of inflammation and oxidative damage. Importantly, the three antioxidant systems were also reported to participate in the regulation of inflammation (Wardyn et al., 2015; Jakobs et al., 2017). For example, Nrf2 loss caused elevated inflammation, and inhibition of inflammation by Nrf2 was closely associated with NF-κB pathway modulation and blocking the upregulation of cytokines such as IL-1β and IL6 (Wardyn et al., 2015). All these results indicated that multi-component TCM such as GHI demonstrated a multi-target and multi-effect response in resisting ischemic stroke, by not only inhibiting the inflammatory response, but also preventing cell apoptosis and oxidative damage, and restoring BBB-related proteins.

Notably, C5AR1 was identified as a vital target in GHI-mediated protection. C5AR1 which belongs to the G protein-coupled receptor (GPCR) family (Mishiro et al., 2012) can be widely expressed by various cells such as neutrophils, monocytes, neurons, astrocytes, and microglia (Brandolini et al., 2019). Research has proved that C5AR1 (also named CD88) played an important role in the regulation of inflammation and neurocognitive functions in ischemic stroke, Alzheimer’s disease, malaria, and neuropathic pain (Moriconi et al., 2014; McDonald et al., 2015; Brandolini et al., 2019). The expression of C5AR1 was significantly increased in MCAO- and OGD-induced modela, and inhibition of C5AR1 by its inhibitors such as DF3016A and PMX53 exhibited a remarkable neuroprotective effect and caused an obvious inhibition in neuro-inflammation and cell apoptosis in primary cortical neurons and a MCAO-induced stroke model (Shi et al., 2017; Brandolini et al., 2019). Notably, deletion of C5AR1 in mice remarkably reduced MCAO surgery-induced infarction volumes and also obviously enhanced neurological scores (Pavlovski et al., 2012)**.** These studies highlight the vital role of C5AR1 in ischemic stroke. Furthermore, knockout of C5AR1 in a mouse-based Alzheimer’s disease model also exhibited less inflammation and inhibited memory deficit in the hippocampal area (Hernandez et al., 2017). Cerebral ischemia-reperfusion initiates complement activation and promotes inflammatory response which exacerbates reperfusion damage after stroke. As the ligand of C5AR1, C5a is a member of the complement system and exhibits multiple inflammatory activities, such as stimulating cells to secret inflammatory factors or recruiting and activating leukocytes (Ducruet et al., 2012). In our study, GHI significantly decreased high IRI-induced C5AR1 and C5A expression *in vivo*. Consistent with this result, GHI prevented cell damage and decreased C5AR1 expression in the C5A-induced Neuro-2a cell model. Notably, HSYA and AG were identified as the major effective components to affect C5AR1. Thus, GHI provided obvious neuroprotection through inhibition of C5AR1 in ischemic stroke.

## Conclusion

In conclusion, consecutive administration of GHI for 6 days after reperfusion remarkably ameliorated inflammation and protected against IRI through decreasing C5AR1. Our study highlighted the benefits of inhibition of inflammation and targeting C5AR1 in the long-term repair of GHI in ischemic stroke. This research provided new insights in the mechanism of GHI-mediated neuroprotection and benefits of its clinical application.

## Data Availability

The datasets presented in this study can be found in online repositories. The RNA-seq data has been uploaded into NCBI and the address is https://www.ncbi.nlm.nih.gov/bioproject/736751.

## Abbreviations

MCAO, middle cerebral artery occlusion; TCM, traditional Chinese medicine; IRI, ischemia/reperfusion injury; GHI, Guhong injection; TTC, 2,3,5-triphenyltetrazolium chloride; AG, aceglutamide; SAAG, s afflower extract and aceglutamide; SA, safflower extract; HSYA, hydroxysafflower yellow A; Neuro-2a, mouse neuroblastoma N2a; 8-OHdG, 8-hydroxy-2′-deoxyguanosine; MMP9, matrix metalloproteinases 9; TIMP1, tissue inhibitor of metalloproteinases 1; C5AR1, C5a anaphylatoxin chemotactic receptor 1; JAM-A, junctional adhesion molecule 1; ICAM-1, intercellular adhesion molecule 1; rCBF, regional cerebral blood flow; H&E, hematoxylin and eosin staining; BBB, blood-brain barrier; ELISA, enzyme linked immunosorbent assay; GO, gene ontology; DEGs, differentially expressed genes; LDH, lactate dehydrogenase; ROS, reactive oxygen species; IF, immunofluorescence staining; I/R, ischemia/reperfusion.
